# Piperine-coated zinc oxide nanoparticles target biofilms and induce oral cancer apoptosis via BCl-2/BAX/P53 pathway

**DOI:** 10.1186/s12903-024-04399-z

**Published:** 2024-06-21

**Authors:** Mohammed Rafi Shaik, Karthikeyan Kandaswamy, Ajay Guru, Haroon Khan, Jayant Giri, Saurav Mallik, Mohd Asif Shah, Jesu Arockiaraj

**Affiliations:** 1 Department of Chemistry, College of Science, King Saud University, P.O. Box 2455, Riyadh, 11451 Saudi Arabia; 2 Department of Cariology, Saveetha Institute of Medical and Technical Sciences, Saveetha Dental College and Hospitals, Saveetha University, Chennai, India; 3 Department of Pharmacy, Abdul wali Khan University Mardan, Mardan, 23200 Pakistan; 4 Department of Mechanical Engineering, Yeshwantrao Chavan College of Engineering, Nagpur, India; 5 Molecular and Integrative Physiological Sciences, Department of Environmental Health, Harvard T. H. Chan School of Public Health, Boston, MA 02115 USA; 6 Department of Pharmacology and Toxicology, University of Arizona, Tucson, AZ 85721 USA; 7 Department of Economics, Kebri Dehar University, 250, Kebri Dehar, Somali Ethiopia; 8 Division of Research and Development, Lovely Professional University, Phagwara, Punjab 144001 India; 9 Toxicology and Pharmacology Laboratory, Department of Biotechnology, Faculty of Science and Humanities, SRM Institute of Science and Technology, Chengalpattu District, Kattankulathur, Tamil Nadu 603203 India

**Keywords:** Piperine, Zinc oxide nanoparticle, Anticancer, Antimicrobial

## Abstract

**Background:**

Dental pathogens play a crucial role in oral health issues, including tooth decay, gum disease, and oral infections, and recent research suggests a link between these pathogens and oral cancer initiation and progression. Innovative therapeutic approaches are needed due to antibiotic resistance concerns and treatment limitations.

**Methods:**

We synthesized and analyzed piperine-coated zinc oxide nanoparticles (ZnO-PIP NPs) using UV spectroscopy, SEM, XRD, FTIR, and EDAX. Antioxidant and antimicrobial effectiveness were evaluated through DPPH, ABTS, and MIC assays, while the anticancer properties were assessed on KB oral squamous carcinoma cells.

**Results:**

ZnO-PIP NPs exhibited significant antioxidant activity and a MIC of 50 µg/mL against dental pathogens, indicating strong antimicrobial properties. Interaction analysis revealed high binding affinity with dental pathogens. ZnO-PIP NPs showed dose-dependent anticancer activity on KB cells, upregulating apoptotic genes BCL2, BAX, and P53.

**Conclusions:**

This approach offers a multifaceted solution to combatting both oral infections and cancer, showcasing their potential for significant advancement in oral healthcare. It is essential to acknowledge potential limitations and challenges associated with the use of ZnO NPs in clinical applications. These may include concerns regarding nanoparticle toxicity, biocompatibility, and long-term safety. Further research and rigorous testing are warranted to address these issues and ensure the safe and effective translation of ZnO-PIP NPs into clinical practice.

**Supplementary Information:**

The online version contains supplementary material available at 10.1186/s12903-024-04399-z.

## Introduction

Ensuring optimal oral health is paramount due to the significant impact of dental pathogens and oral cancer on overall well-being [1]. Dental pathogens are associated with various oral diseases such as tooth decay, gum disease, and oral infections. Left untreated, these conditions can lead to pain, discomfort, tooth loss, and even systemic health issues due to the potential for bacteria to enter the bloodstream [2]. Moreover, oral cancer, which can arise from multiple factors including tobacco use, alcohol consumption, and HPV infection, presents a grave concern [3]. *S. aureus* is associated with dental caries, periodontal disease, and oral abscesses, posing challenges due to its ability to produce toxins and antibiotic resistance [4]. *S. mutans* is a primary cause of dental caries, producing acids that erode tooth enamel and forming biofilms that contribute to plaque formation [5]. *E. faecalis* is implicated in persistent root canal infections and treatment failures, often displaying resistance to conventional antimicrobial agents [6]. *C. albicans* can lead to oral thrush, particularly in immunocompromised individuals, with the potential for systemic infections [7]. Understanding the mechanisms of pathogenesis and host-pathogen interactions of these dental pathogens is essential for developing targeted therapies and preventive strategies to mitigate their impact on oral health and overall well-being.

Oral cancer presents significant challenges stemming from late diagnosis, high metastatic potential, treatment complexities, and frequent recurrence. Dental pathogens interact with oral cancer through multifaceted mechanisms [8]. Firstly, chronic inflammation induced by pathogens creates a microenvironment conducive to tumor development. These pathogens release pro-inflammatory cytokines and activate immune cells, perpetuating inflammation and facilitating carcinogenesis [9–12]. Secondly, certain dental pathogens produce genotoxic substances, such as reactive oxygen species (ROS), causing DNA damage and genomic instability in oral epithelial cells [13]. This genetic damage can lead to the initiation and progression of cancerous lesions. Additionally, dysbiosis of the oral microbiome, often seen in periodontal disease, may contribute to oral cancer development through interactions with host immune responses and signaling pathways. Furthermore, dental pathogens may directly influence oncogenic pathways within oral cells, promoting cell proliferation and survival [14]. In addition to understanding the pathogenesis of oral cancer, it is crucial to discuss its diagnosis and treatment. Diagnosis typically involves a combination of clinical examination, imaging techniques such as computed tomography (CT) scans and magnetic resonance imaging (MRI), and biopsy for histopathological analysis [15]. Treatment options vary depending on the stage and location of the tumor but may include surgery, radiation therapy, chemotherapy, targeted therapy, and immunotherapy [16]. Multidisciplinary approaches involving oncologists, surgeons, dentists, and other healthcare professionals are often necessary to provide comprehensive care to patients with oral cancer.

Piperine (PIP), a naturally occurring alkaloid found in black pepper and other plants, possesses potent antimicrobial effects against various pathogens, including bacteria and fungi, making it an attractive candidate for oral health applications [17]. Meanwhile, Zinc oxide nanoparticle (ZnO NPs) have demonstrated significant anticancer activity against cancer cells, inhibiting their proliferation and inducing apoptosis [18]. Previous studies have explored the use of ZnO NPs coated with cinnamic acid and curcumin for antimicrobial activity against dental pathogens and targeting the BCL-2/BAX/P53 pathway to enhance apoptosis in oral squamous cells [19, 20]. Coating ZnO NPs with PIP could potentially enhance their efficacy and selectivity against oral cancer cells while minimizing off-target effects. This unique combination has not been previously reported in the literature, thus representing a novel approach in the field. Combining PIP and ZnO NPs can potentially enhance their individual therapeutic effects against dental pathogens and oral cancer. PIP may augment the antimicrobial efficacy of ZnO NPs by disrupting the cell membranes of dental pathogens, thereby increasing their susceptibility to NPs-induced damage. Additionally, PIP ability to modulate intracellular signaling pathways involved in cancer cell growth and survival may potentiate the anticancer effects of ZnO NPs. In this study, we combined PIP and ZnO NPs for their targeted delivery of formulation on the surface of dental pathogen receptors and oral cancer cells to enhance their activity and efficacy in oral medicine, thereby offering a promising strategy for addressing both infectious and malignant oral conditions.

## Materials and methods

### Synthesis and characterization of ZnO-PIP NPs

A 10 mg/mL concentrated solution of PIP was prepared by dissolving 50 mg of PIP powder in 5 mL of ethanol. ZnO NPs were synthesized by the precipitation method. Zinc acetate dihydrate (Zn(CH_3_COO)_2_·2 H_2_O) (0.1 M, 20 mL) was dissolved in the PIP solution under constant stirring at room temperature for 1 h. Subsequently, sodium hydroxide (NaOH) solution (0.1 M) was added dropwise to the zinc acetate solution until the pH reached approximately 10. The resulting mixture was stirred for an additional 2 h to ensure complete reaction. The green synthesis approach facilitated the formation of plant compound-ZnO NPs, which were then centrifuged at 8000 rpm for 10 min, washed with distilled water to remove impurities, and finally dried at 60 °C for 12 h under vacuum. NPs suspensions in distilled water were scanned in the wavelength range of 200–800 nm using a UV-Vis spectrophotometer to obtain the absorption spectrum, which provided insights into the bandgap energy of the NPs. The morphology and size distribution of the synthesized NPs were examined using a scanning electron microscope (SEM). Powder samples were loaded onto a sample holder and scanned at a 2θ range of 10°-80° with a step size of 0.02°. X-ray Diffraction analysis (XRD) analysis provided information regarding the crystallographic phases and crystallite size of the NPs. Chemical bonding and functional groups present in the synthesized NPs were investigated using Fourier-transform infrared spectroscopy (FTIR) and Energy dispersive X-ray analysis (EDAX) [21]. Maintaining precise control over reaction conditions like temperature, pH, and reaction time is crucial for reproducibility. Optimizing these parameters for larger batches is necessary. Additionally, sourcing high-quality, cost-effective raw materials like zinc acetate dihydrate and piperine in bulk while ensuring consistency poses logistical challenges.

### 2,2-diphenyl-1-picrylhydrazyl (DPPH)

The DPPH assay is based on the ability of antioxidants to donate hydrogen atoms or electrons to neutralize the DPPH radical, a stable free radical with an unpaired electron. The DPPH assay was performed to evaluate the antioxidant activity of the test samples. Briefly, various concentrations (5 µg/mL, 25 µg/mL, 50 µg/mL, & 100 µg/mL) of the ZnO-PIP NPs were prepared. A solution of DPPH radical (0.1 mM) was prepared by dissolving DPPH in ethanol. Aliquots (100 µL) of the ZnO-PIP NPs at different concentrations were mixed with 900 µL of DPPH solution and incubated in the dark at room temperature for 30 min. The absorbance of the resulting solution was measured at 517 nm using a UV-Vis spectrophotometer [22].

### 2,2′-azino-bis-(3-ethylbenzothiazoline-6-sulfonic) (ABTS)

The ABTS assay measures the ability of antioxidants to scavenge ABTS^•+^ radicals, which are formed by the oxidation of ABTS with potassium persulfate. The ABTS^•+^ radical cation was generated by mixing ABTS solution (7 mM) with potassium persulfate (2.45 mM) and allowing the mixture to stand in the dark at room temperature for 16 h. The resulting ABTS^•+^ solution was diluted with ethanol to obtain an absorbance of 0.70 ± 0.02 at 734 nm. Various concentrations of the ZnO-PIP NPs (5 µg/mL, 25 µg/mL, 50 µg/mL, & 100 µg/mL) were prepared. Aliquots (100 µL) of the Zno-PIP NPs at different concentrations were mixed with 900 µL of the diluted ABTS^•+^ solution and incubated in the dark at room temperature for 6 min. The absorbance of the resulting solution was measured at 734 nm using a UV-Vis spectrophotometer [23].

### Minimal inhibitory concentration (mic) assay

The MIC assay was performed to determine the lowest concentration of the ZnO-PIP NPs required to inhibit the visible growth of the dental pathogens (*S. aureus*, *S. mutans*, *E. faecalis*, *C. albicans*). Each concentration of the (100 µL) was ZnO-PIP added to individual wells of a sterile microtiter plate. To each well, 100 µL of inoculum containing a standardized suspension of the dental pathogens at a concentration of approximately 1–5 × 10^5^ colony-forming units per milliliter (CFU/mL) was added. The microtiter plate was then covered and incubated at the appropriate temperature for the specific microorganism (e.g., 37 °C for bacteria, 25 °C for fungi) for 18–24 h. Following incubation, the growth of the microorganism in each well was visually inspected, and the MIC was defined as the lowest concentration of the test compound that completely inhibited visible growth, indicated by the absence of turbidity or visible colonies [24].

### Well diffusion method for zone of inhibition

The well diffusion method was employed to evaluate the antimicrobial activity of the ZnO-PIP NPs against a dental pathogens. Nutrient agar plates were prepared and inoculated with a standardized suspension of the test microorganism by streaking the agar surface using a sterile swab to achieve a lawn of growth. Wells were created on the agar plates using a sterile cork borer or a commercially available well puncher. The 20 µL of different concentration of Zn-PIP NPs were added to separate wells on the agar plates. Additionally, wells containing the standard antimicrobial agent of known activity (positive control) were also created on the agar plates. The plates were then incubated at the appropriate temperature for the specific microorganism (e.g., 37 °C for bacteria, 25 °C for fungi) for 18–24 h. Following incubation, the plates were examined, and the diameter of the clear zone surrounding each well was measured using a calibrated ruler. The diameter of the zone of inhibition was recorded in millimeters (mm) as an indicator of the antimicrobial activity of the ZnO-PIP NPs.

### Docking studies

AutoDock is a widely used software for molecular docking studies that predicts the binding affinity values (kcal/mol) between the ligand and receptor. The 2D structure of the PIP compound was obtained from the PubChem database (https://pubchem.ncbi.nlm.nih.gov/ ) and converted into 3D format using software such as PyMOL. The receptor structures of dental pathogens were obtained from the Protein Data Bank (PDB) (https://www.rcsb.org/) and prepared for docking studies using Autodock tool ver 1.5.7. Hydrogens, charges, and appropriate bonds were added to both ligand and receptor structures. The prepared ligand and receptor structures were imported into AutoDock Tools. The receptor was defined as a rigid structure, while the ligand was set as flexible to explore different conformations. The binding site of the receptor was defined based on the active site residues or known binding pockets. Parameters such as grid box size, spacing, and search parameters were set according to the specifications of AutoDock. Lamarckian genetic algorithm parameters were adjusted to ensure efficient exploration of the conformational space. Finally after the analysis, the interaction between the ligand and receptor was visulaized in the Discovery studio visualizer software [25].

### Anticancer assay

KB cells (human epithelial carcinoma cells) were cultured in Dulbecco’s Modified Eagle’s Medium (DMEM) supplemented with 10% fetal bovine serum (FBS) and 1% penicillin-streptomycin in a humidified incubator at 37 °C with 5% CO_2_. Upon reaching 70–80% confluency, the cells were treated with various concentrations of the ZnO-PIP NPs were treated to cells. After incubation with the ZnO-PIP NPs for 24 h, the viability of KB cells was assessed using the 3-(4,5-dimethylthiazol-2-yl)-2,5-diphenyltetrazolium bromide (MTT) assay. Briefly, the culture medium was removed, and the cells were washed with phosphate-buffered saline (PBS). MTT solution (5 mg/mL in PBS) was added to each well, and the cells were further incubated for 3 h at 37 °C. Following incubation, the MTT solution was aspirated, and dimethyl sulfoxide (DMSO) was added to solubilize the formazan crystals. The absorbance of the resulting solution was measured at 570 nm using a microplate reader [26].

### Apoptosis gene expression

The expression levels of specific genes can be quantified by measuring the amount of amplified cDNA product, providing valuable insights into gene regulation under different experimental conditions. Total RNA was extracted from ZnO-PIP treated KB cells using the Trizol reagent according to the manufacturer’s instructions. Briefly, cells were lysed in Trizol reagent, and chloroform was added to separate the RNA-containing aqueous phase. The RNA was precipitated with isopropanol, washed with ethanol, and resuspended in RNase-free water. The quality and quantity of RNA were assessed using a spectrophotometer. First-strand cDNA synthesis was performed using a reverse transcription kit according to the manufacturer’s protocol. Briefly, RNA samples were incubated with a mixture containing reverse transcriptase enzyme, random primers, dNTPs, and RNase inhibitor at 37 °C for 1 h to synthesize cDNA. The reaction was then terminated by heating at 95 °C for 5 min, and the cDNA was stored at -20 °C until further analysis. Quantitative RT-PCR (qRT-PCR) was performed to analyze the expression levels of target genes in ZnO-NPs treated KB cells. Gene-specific primers and list of abbreviation were given in Table 1 and E-supplement Table 1. The amplification conditions included an initial denaturation step at 95 °C for 5 min, followed by 40 cycles of denaturation at 95 °C for 30 s, annealing for 30 s, and extension at 72 °C for 30 s. The mRNA expression levels were normalized to a housekeeping gene, such as GAPDH, and calculated using the 2^−ΔΔCt^ method [27].

**Table 1:** Primers used for gene expression

Gene	Forward primer (5´-3´)	Reverse primer (5´-3´)	Reference
GAPDH	GCCAAAAGGGTCATCATCTCTGC	GGTCACGAGTCCTTCCACGATAC	[28]
BCL-2	GACGACTTCTCCCGCCGCTAC	CGGTTCAGGTACTCAGTCATCCAC	[28]
BAX	AGGTCTTTTTCCGAGTGGCAG	GCGTCCCAAAGTAGGAGAGGAG	[28]
P53	ACATGACGGAGGTTGTGAGG	TGTGATGATGGTGAGGATGG	[29]

### Statistical analysis

Statistical analysis was conducted to assess the significance of differences among various concentrations of ZnO-PIP NPs treatment group and the control (untreated) using a one-way analysis of variance (ANOVA), followed by post-hoc multiple comparisons (Tukey’s test). Results were deemed statistically significant at *p* < 0.05. Data are presented as mean ± standard deviation, with each experiment performed in triplicate.

## Results

### Synthesis and characterization of ZnO-PIP NPs

**Fig. 1:**
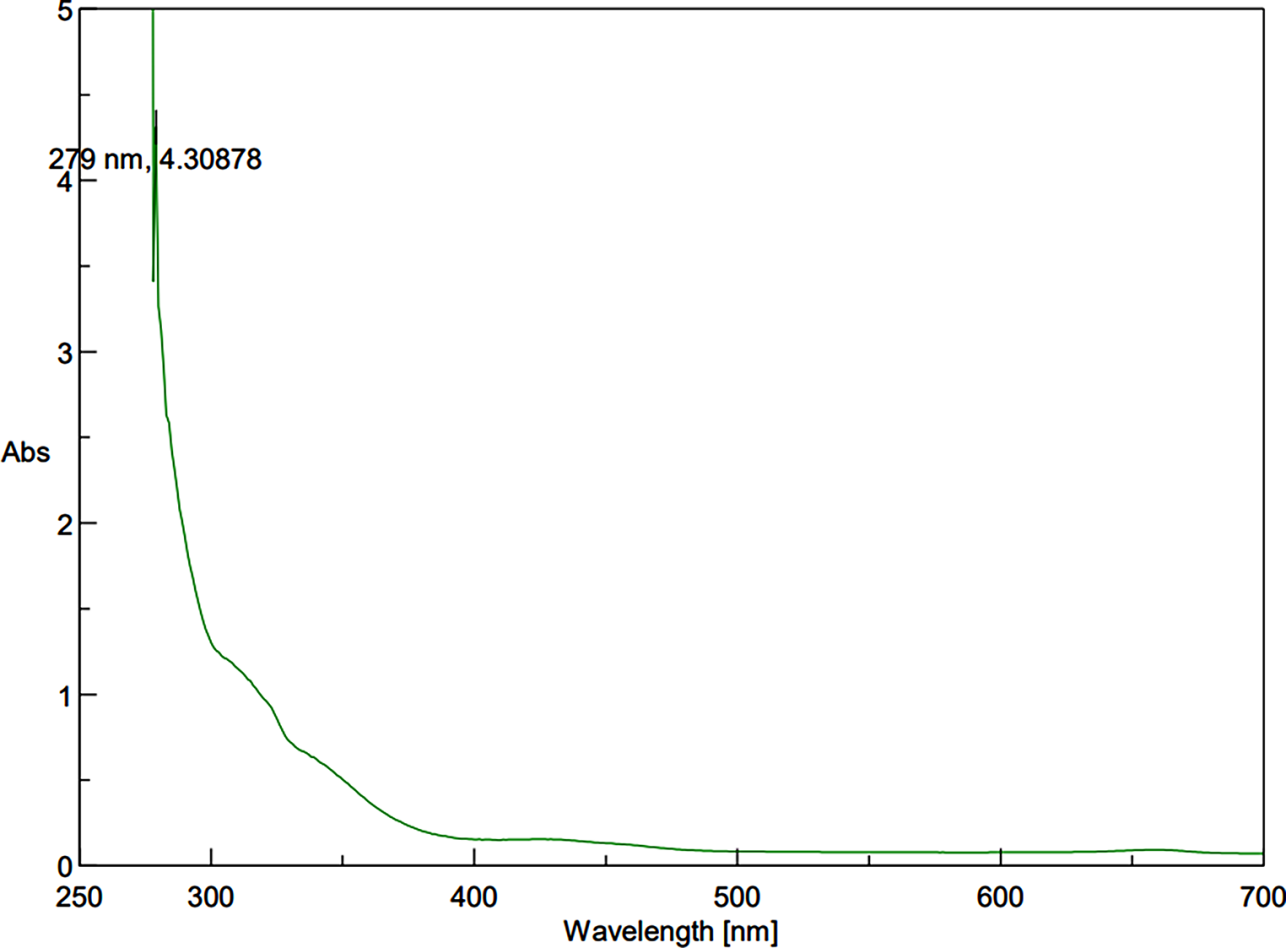
UV absorption spectra of synthesized ZnO-PIP NPs

**Fig. 2:**
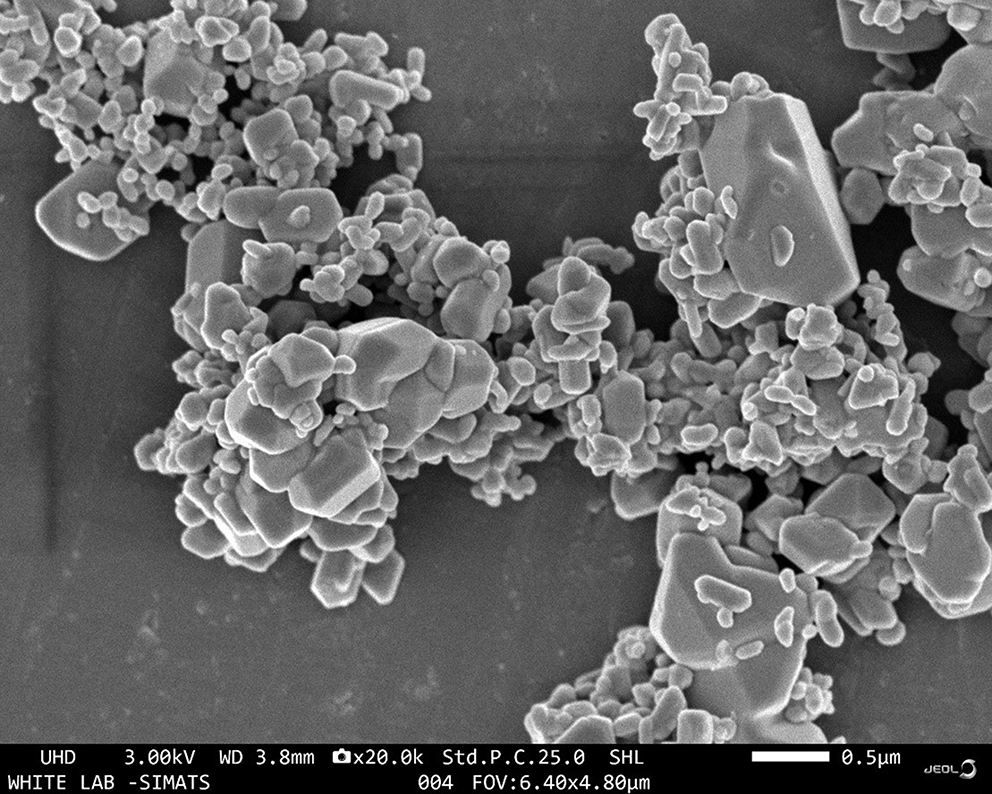
SEM images revealed crystal-like structure in the ZnO-PIP NPs

**Fig. 3:**
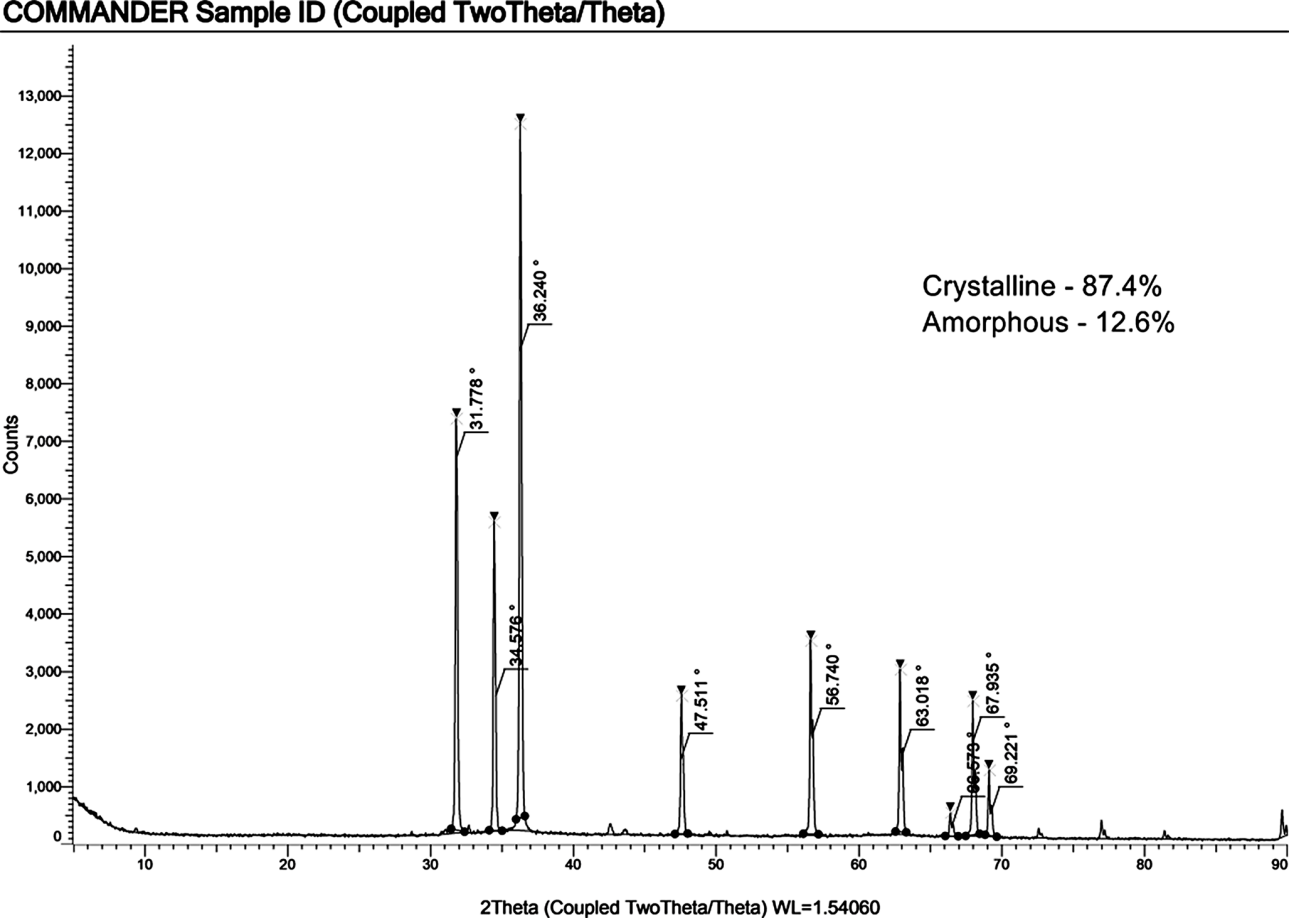
XRD characterization of ZnO-PIP NPs

**Fig. 4:**
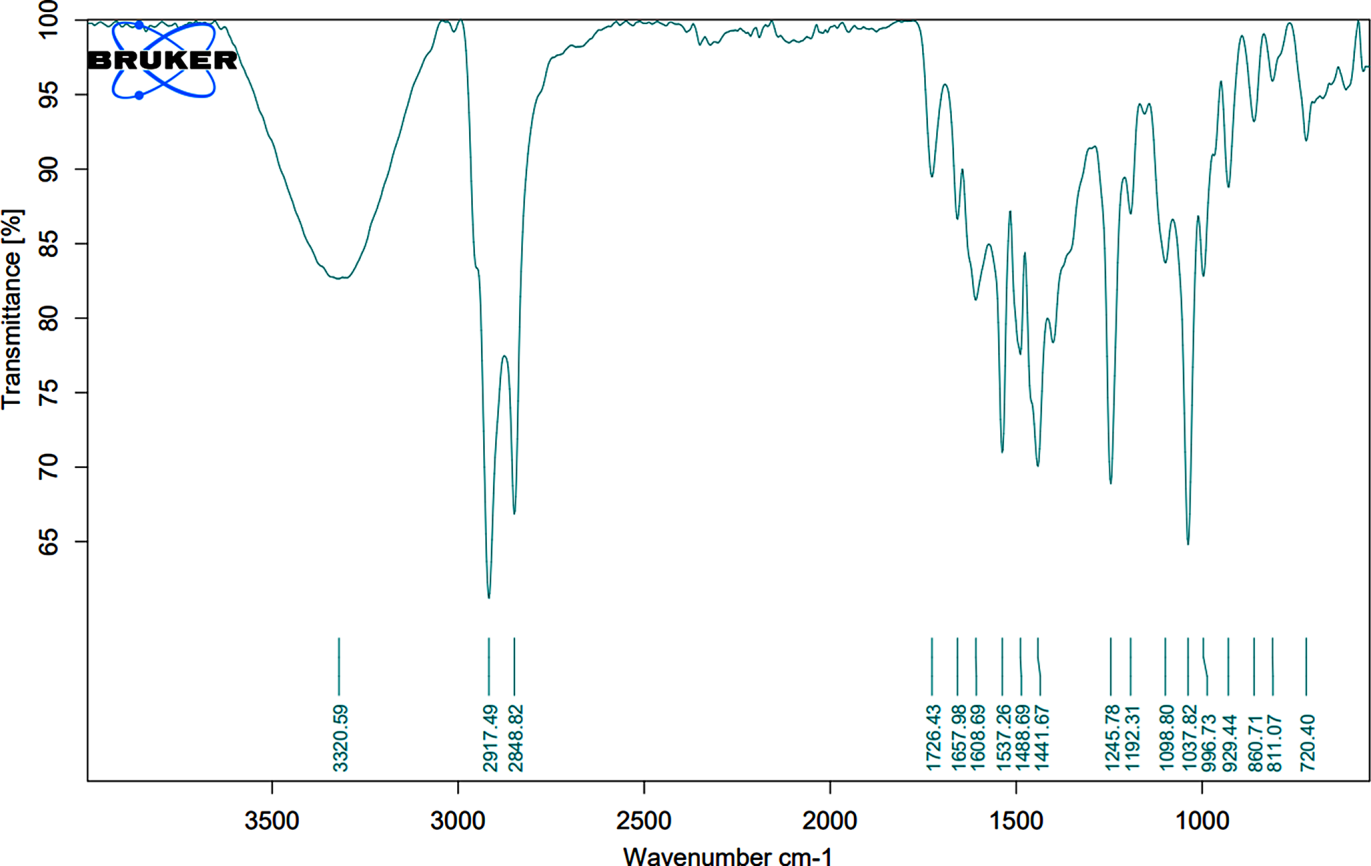
FTIR characterization of ZnO-PIP NPs

**Fig. 5:**
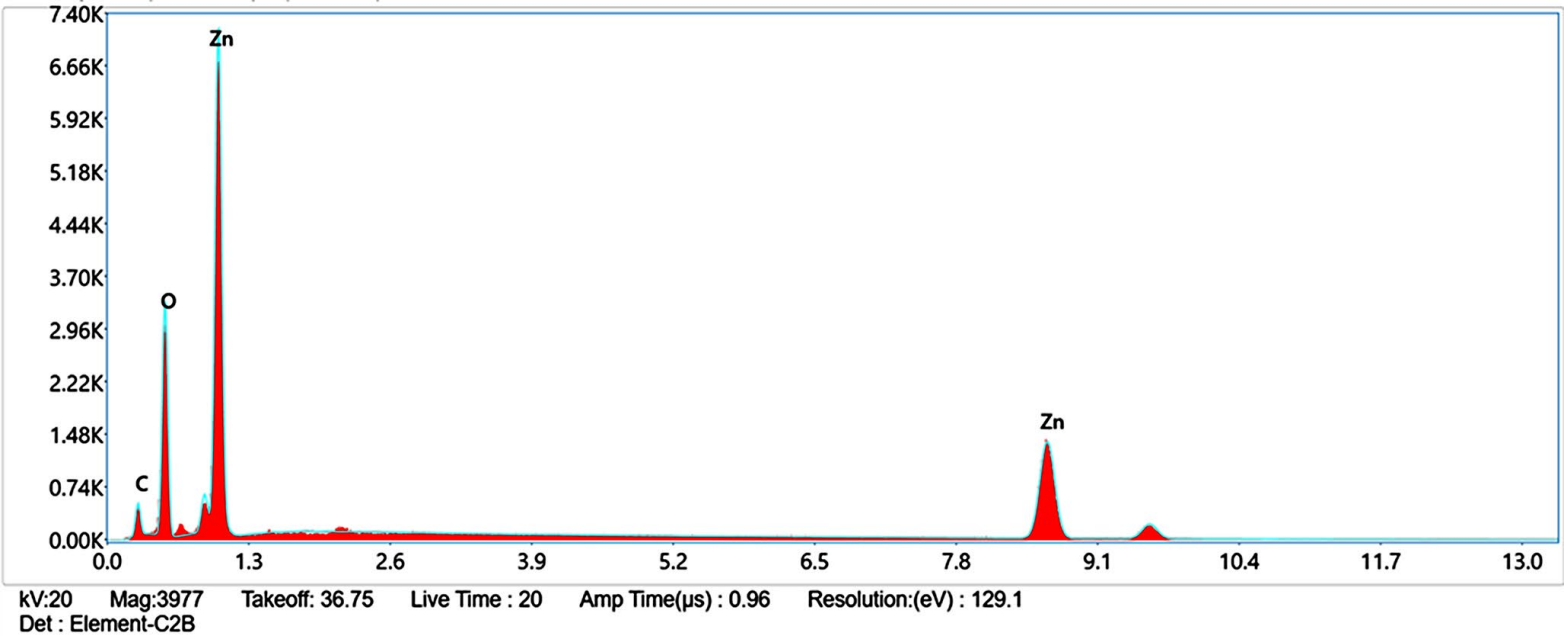
EDAX analysis of ZnO-PIP NPs

The UV-Vis spectroscopy analysis of ZnO-PIP NPs reveals a prominent absorption peak at 279 nm with an absorbance value of 4.30878 (Fig. 1). This absorption peak corresponds to the electronic transitions occurring within the ZnO NPs. Specifically, the observed peak at 279 nm is attributed to the bandgap absorption of ZnO, which is characteristic of its semiconductor nature. The absorption of photons at this wavelength promotes electrons from the valence band to the conduction band, indicating the excitation of charge carriers within the NPs. The SEM analysis of ZnO-PIP NPs reveals a complex morphology characterized by the presence of crystallized NPs with varying sizes ranging below 500 nm (Fig. 2). The NPs exhibit a tendency to agglomerate, forming clusters of irregular shapes. This agglomeration may be attributed to the strong interparticle interactions or the surface chemistry of the NPs mediated by the presence of PIP. Despite the agglomeration, individual NPs display relatively smooth surfaces with occasional surface irregularities. The SEM images also hint at the presence of crystalline structures, with some NPs showing facets indicative of crystal faces, although further confirmation through techniques like X-ray diffraction would be necessary to elucidate the crystallographic nature of the NPs. The XRD analysis of ZnO-PIP NPs exhibits a crystalline phase comprising 87.4% of the sample, alongside an amorphous phase constituting 12.6% of the material. The XRD pattern reveals several distinct peaks at various 2θ values, indicative of the crystalline nature of the NPs. Specifically, the observed peaks at 2θ values of 31.77°, 34.57°, 36.24°, 47.51°, 56.74°, 63.01°, 67.93°, and 69.22° correspond to different planes of the ZnO crystal lattice (Fig. 3). These peaks can be indexed to the hexagonal wurtzite crystal structure of ZnO, with the (100), (002), (101), (102), (110), (103), (200), and (112) planes, respectively. The presence of well-defined peaks indicates the high crystallinity and purity of the ZnO NPs, with minimal presence of impurities or amorphous phases. The FTIR observed peaks at 3320.59 cm^− 1^, 2917.49 cm^− 1^, and 2848.82 cm^− 1^ correspond to stretching vibrations of hydroxyl (OH) groups and aliphatic C-H bonds, respectively, indicating the presence of surface hydroxyl groups and organic moieties possibly originating from the PIP capping agent (Fig. 4). The peak at 1762.43 cm^− 1^ suggests the presence of carbonyl (C = O) stretching vibrations, indicative of functional groups present in PIP. Peaks observed at 1657.98 cm^− 1^, 1608.69 cm^− 1^, and 1537.26 cm^− 1^ correspond to aromatic C = C stretching vibrations, further confirming the presence of aromatic rings in PIP. Additionally, peaks at 1488.69 cm^− 1^ and 1441.67 cm^− 1^ are associated with C-H bending vibrations in the aromatic ring, while peaks at 1245.78 cm^− 1^ and 1192.31 cm^− 1^ suggest C-O stretching vibrations, possibly from the Zn-O bond in ZnO-NPs or functional groups in PIP. Furthermore, peaks at 1098.80 cm^− 1^, 1037.82 cm^− 1^, and 996.73 cm^− 1^ correspond to ZnO stretching vibrations, indicating the presence of ZnO. Peaks at lower wavenumbers, such as 860.71 cm^− 1^, 811.07 cm^− 1^, and 720.4 cm^− 1^, may be attributed to lattice vibrations or structural features of ZnO NPs. The EDAX analysis detect the presence of carbon signals is consistent with the organic nature of the PIP capping agent used during NPs synthesis (Fig. 5). The prominent peaks corresponding to Zn and O confirm the presence of ZnO-NPs in the sample. The elemental composition analysis suggests that the NPs are predominantly composed of Zn and O, in accordance with the expected composition of ZnO.

### Antioxidant activity activity of ZnO-PIP NPs

**Fig. 6:**
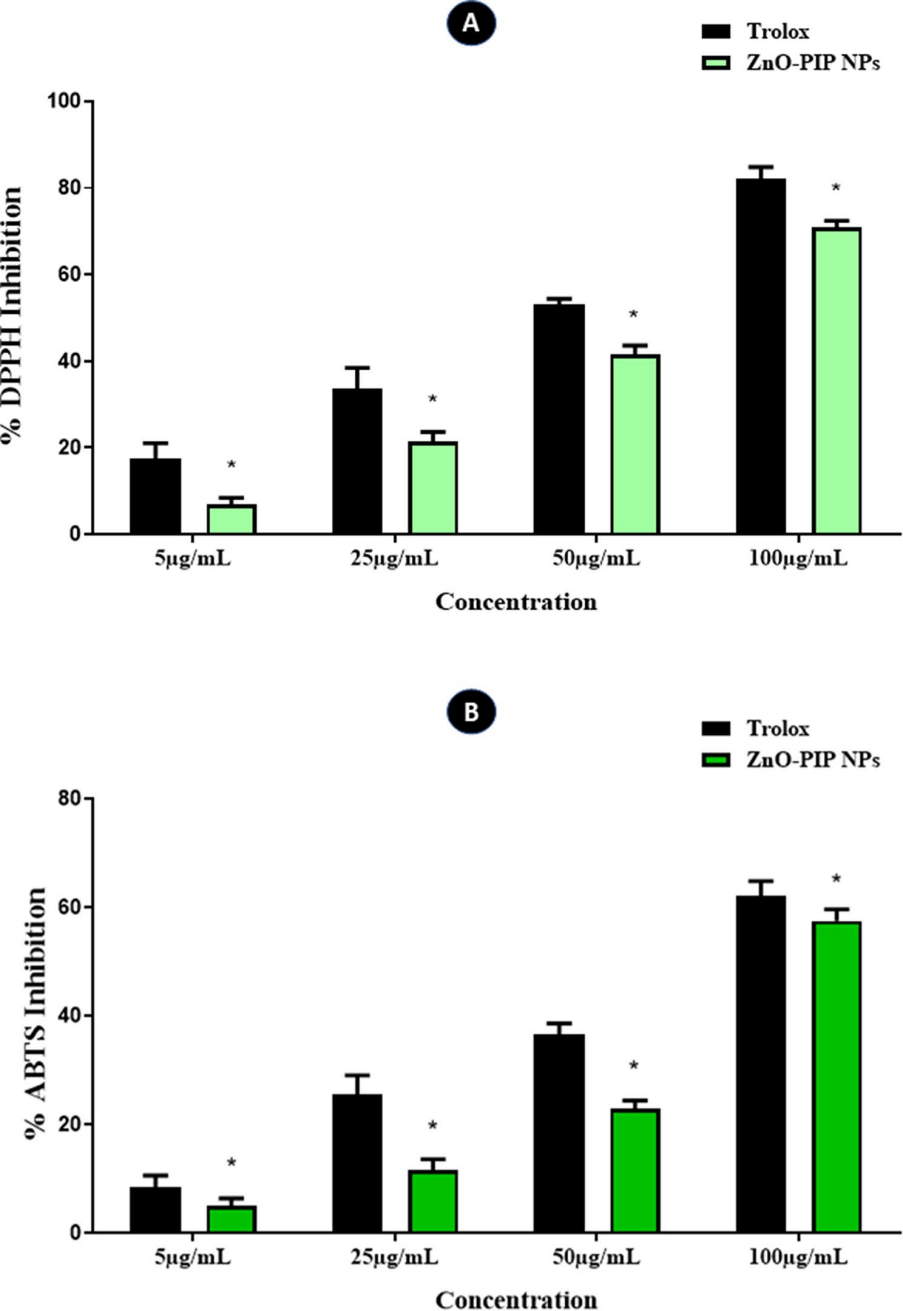
The antioxidant potential of ZnO-PIP NPs was assessed at concentrations of 5 µg/mL, 25 µg/mL, 50 µg/mL, and 100 µg/mL using both DPPH and ABTS assays. Trolox served as the positive control. The asterisk (*) indicates statistical significance (*p* < 0.05) compared to the control group. Results are presented as the mean ± standard deviation of three independent experiments

The results of the DPPH assay (Fig. 6A) display a dose-responsive scavenging impact of ZnO-PIP NPs on DPPH free radicals. The scavenging activity percentage increases with concentrations of 5 µg/mL, 25 µg/mL, 50 µg/mL, and 100 µg/mL, showing respective values of 7%, 22%, 42%, and 71%. Meanwhile, at concentrations of 100 µg/mL, the antioxidant potential of ZnO-PIP NPs demonstrates comparable effectiveness to the Trolox.

The results from the ABTS assay (Fig. 6B) indicate a dose-dependent scavenging effect of ZnO-PIP NPs on ABTS radicals. The reduction in absorbance, signifying the scavenging of ABTS radicals, was observed as ZnO-PIP NPs facilitated the conversion of ABTS to its radical cation (ABTS•+). With increasing concentrations of ZnO-PIP NPs (5 µg/mL, 25 µg/mL, 50 µg/mL, and 100 µg/mL), there was a significant rise in the percentage of ABTS radical scavenging, with values of 5%, 12%, 23%, and 58%, respectively. Moreover, the scavenging of ABTS radicals by ZnO-PIP NPs at concentrations of 100 µg/mL exhibited better activity.

### Antimicrobial activity of ZnO-PIP NPs against dental pathogens

**Fig. 7:**
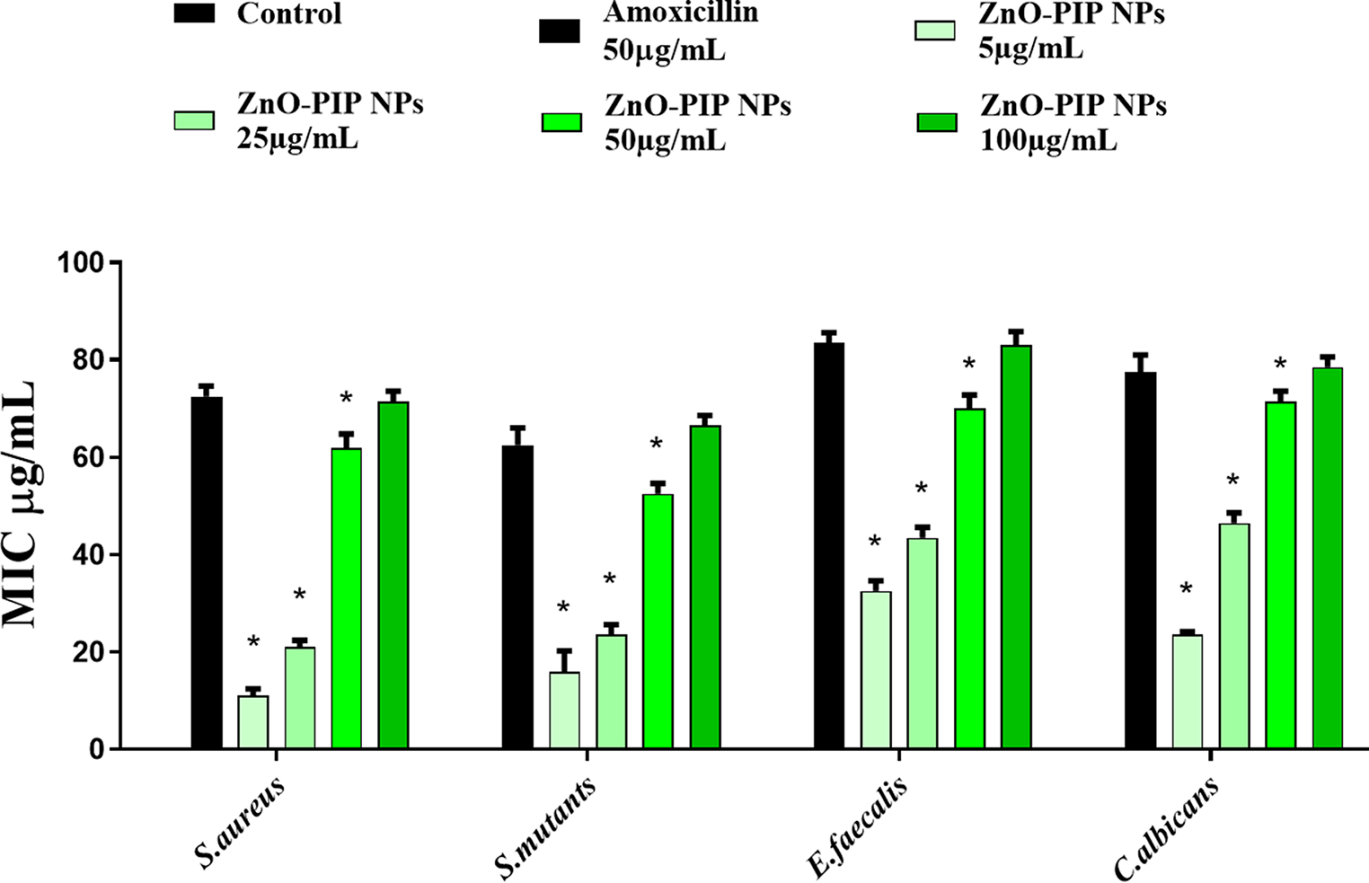
The MIC of ZnO-PIP NPs was evaluated against dental pathogens including *S. aureus*, *S. mutans*, *E. faecalis*, and *C. albicans* at various concentrations. Amoxicillin served as the positive control. The asterisk (*) denotes statistical significance (*p* < 0.05) compared to the control. Results are expressed as the mean ± standard deviation from three distinct experiments

ZnO-PIP NPs consistently showed effective MIC values against the dental pathogens tested, including *S. aureus*, *S. mutans*, *E. faecalis*, and *C. albicans*, all exhibiting susceptibility at 50 µg/mL. This suggests that concentrations of 50 µg/mL and higher effectively inhibited the growth of these pathogens (Fig. 7). Additionally, the zone of inhibition assay demonstrated the antimicrobial activity of ZnO-PIP NPs against all tested dental pathogens. At 50 µg/mL, the zones of inhibition ranged from 8, 7, 12, and 11 mm, and at 100 µg/mL, they increased further, ranging from 15, 14, 18, and 16 mm for *S. aureus*, *S. mutans*, *E. faecalis*, and *C. albicans*, respectively (Table 2). The ZnO-PIP NPs at 50 µg/mL showed slightly smaller zones of inhibition compared to the positive control, amoxicillin (50 µg/mL). But at the same time ZnO-PIP NPs at 100 µg/mL, their antimicrobial activity significantly improved, surpassing that of amoxicillin for all tested pathogens.

**Table 2:** Zone of inhibition of different dental pathogens by ZnO-PIP NPs. The different groups such as Control, Amoxicillin (50 µg/mL), ZnO-PIP NPs at 50 µg/mL, and ZnO-PIP NPs at 100 µg/mL. Zone of inhibition was measured at mm

Zone of Inhibition (mm)				
Bacterial Strain	Control	Amoxicillin 50 µg/mL	ZnO-PIP 50 µg/mL	ZnO-PIP 100 µg/mL
*Staphylococcus aureus*	-	16	8	15
*Streptococcus mutans*	-	15	7	14
*Enterococcus faecalis*	-	17	12	18
*Candida albicans*	-	15	11	16

### PIP interaction with dental pathogen biofilm receptor

**Fig. 8:**
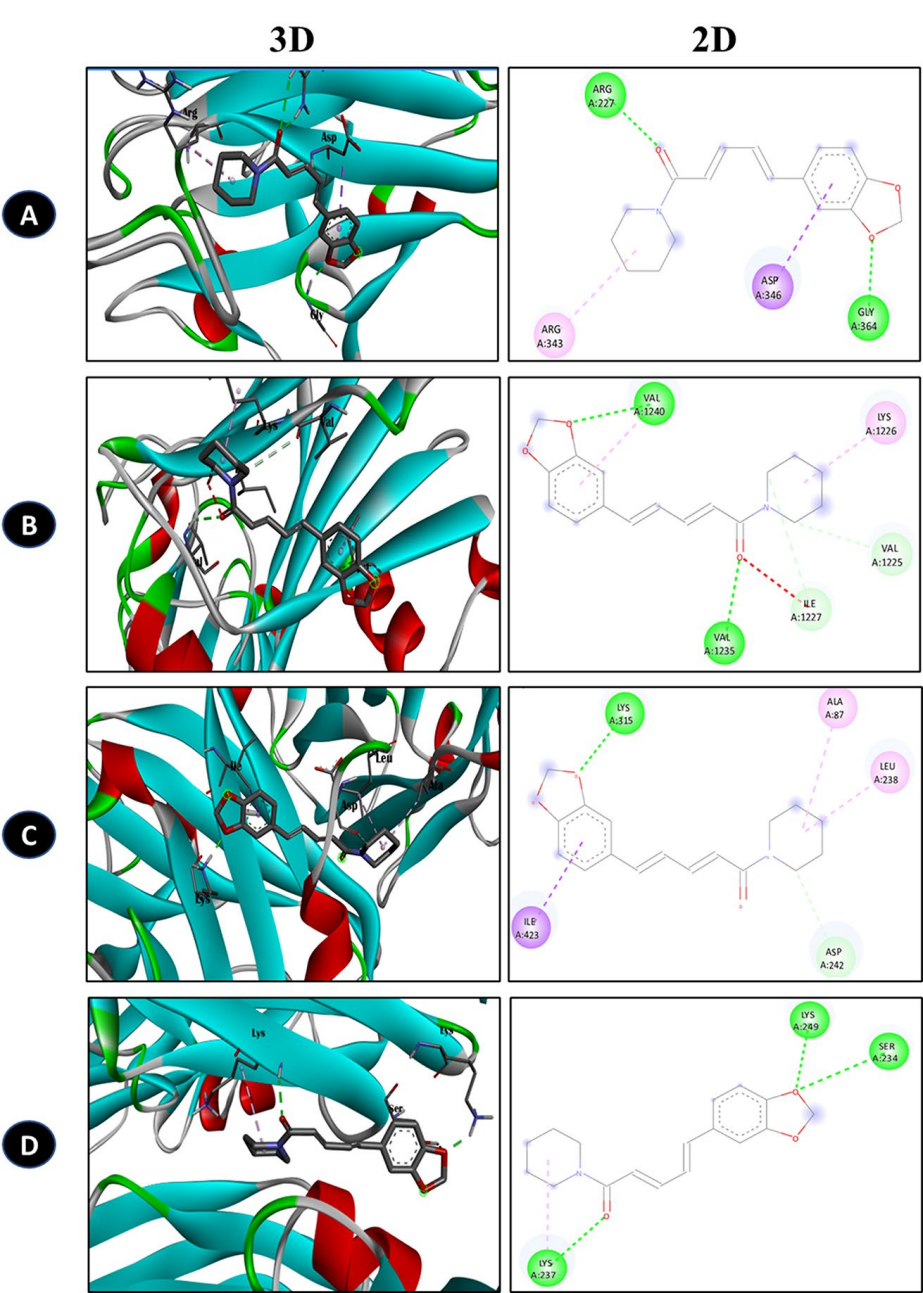
3D and 2D representative images of PIP and dental pathogen receptor interactions. (**A**) *Staphylococcus aureus* surface protein, G (**B**) Antigen I/II carboxy-terminus, (**C**) Enterococcal surface protein, (**D**) ALS3. The interaction are observed with amino acids such as LYS (Lysine), GLY (Glycine), ALA (Alanine), ARG (Arginine), VAL (Valine), ILE (Isoleucine), and SER (Serine)

The molecular docking investigation unveiled promising interactions between PIP and the receptor proteins of four dental pathogens: *S. aureus*, *S. mutans*, *E. faecalis*, and *C. albicans* (Fig. 8). Notably, PIP exhibited higher binding affinity values with the pathogen receptors, indicating its potential as a therapeutic agent against dental infections (Table 3). Specifically, in the docking analysis with *S. aureus* surface protein G (PDB ID: 7SMH), PIP demonstrated a binding affinity of -6.77 kcal/mol. This interaction involved crucial amino acids such as ARG, ASP, GLY, and ARG suggesting a potential disruption of the surface protein and consequently hindering *S. aureus* pathogenicity. Similarly, for *S. mutans*, the docking analysis with Antigen I/II carboxy-terminus (PDB ID: 3QE5) yielded a binding affinity of -6.1 kcal/mol. Interactions with amino acids VAL, VAL, LYS, ILE, and VAL indicated that PIP might disrupt the function of Antigen I/II carboxy-terminus, thereby affecting *S. mutans* pathogenicity. Regarding *E. faecalis*, the docking analysis with Enterococcal surface protein (PDB ID: 6ORI) revealed a binding affinity of -8.2 kcal/mol, with interactions involving amino acids ILE, LYS, ALA, and LEU. This suggests that PIP could potentially interfere with this protein, thereby impeding biofilm formation and attenuating *E. faecalis* pathogenicity. Lastly, in the docking analysis with *C. albicans* ALS3 (PDB ID: 4LEE), PIP exhibited a binding affinity of -7.6 kcal/mol, with interactions involving amino acids LYS, LYS, and SER. This indicates that PIP might interfere with the function of ALS3, an essential protein involved in adherence to host surfaces, thereby affecting biofilm formation, a critical step in *C. albicans* pathogenicity.

**Table 3:** Amino acid and Binding affinity value of the p interaction with different dental pathogen receptors. LYS (Lysine), GLY (Glycine), ALA (Alanine), ARG (Arginine), VAL (Valine), ILE (Isoleucine), and SER (Serine)

	Receptor (PDB ID)	Ligand	Binding affinity (kcal/mol)	Amino acid interaction
1.	*S. aureus: Staphylococcus aureus* surface protein G (7SMH)	Piperine	-6.7	ARG, ASP, GLY, and ARG
2.	*S. mutans*: Antigen I/II carboxy-terminus (3QE5)	Piperine	-6.1	VAL, VAL, LYS, ILE, and VAL
3.	*E. faecalis*: Enterococcal surface protein (6ORI)	Piperine	-8.2	ILE, LYS, ALA, and LEU
4.	*C. albicans*: ALS3 (4LEE)	Piperine	-7.6	LYS, LYS, and SER

### Anticancer activity of ZnO-PIP NPs

**Fig. 9:**
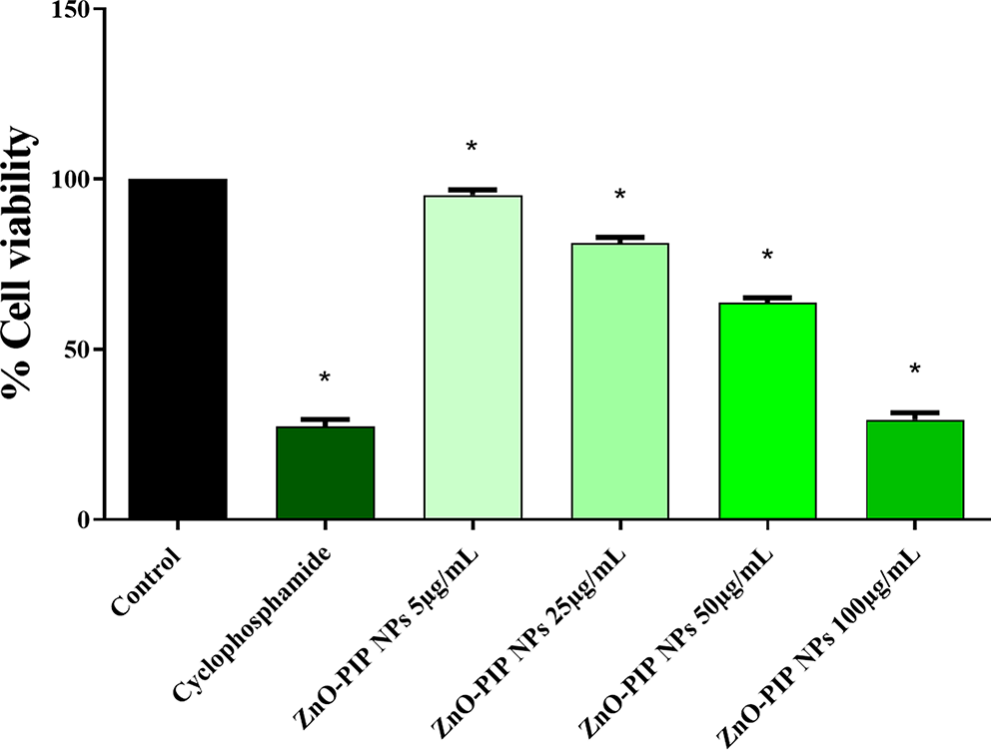
The MTT assay was employed to investigate the in-vitro anticancer potential of ZnO-PIP NPs on KB cells. Various experimental groups were included: untreated control cells **(A)**, a positive control group treated with cyclophosphamide (50 µg/mL) **(B)**, and ZnO-PIP nanoparticles at concentrations of 5 µg/mL, 25 µg/mL, 50 µg/mL, and 100 µg/mL **(C, D, E, & F)**. A graphical representation **(G)** illustrates the percentage of cell viability in KB cancer cells at different concentrations of ZnO-PIP NPs. The asterisk (*) denotes statistical significance (*p* < 0.05) compared to the control. Results are presented as the mean ± standard deviation from three independent experiments

The results depicted in Fig. 9 highlight notable changes in KB cell viability in response to increasing concentrations of ZnO-PIP NPs. At a lower concentration of 5 µg/mL, there was only minimal impact on cell viability following 24 h of exposure. In contrast, the group treated with Cyclophosphamide exhibited a significant reduction in cell viability, dropping to 27%. However, at higher concentrations, particularly 100 µg/mL, a marked decrease in KB cell viability (29%) was evident, resembling the cell viability observed in the positive control group. These findings suggest a concentration-dependent anticancer effect of ZnO-PIP NPs on KB cells, indicating potential implications for oral cancer treatment.

### Regulating apoptosis signaling pathways in oral cancer

**Fig. 10:**
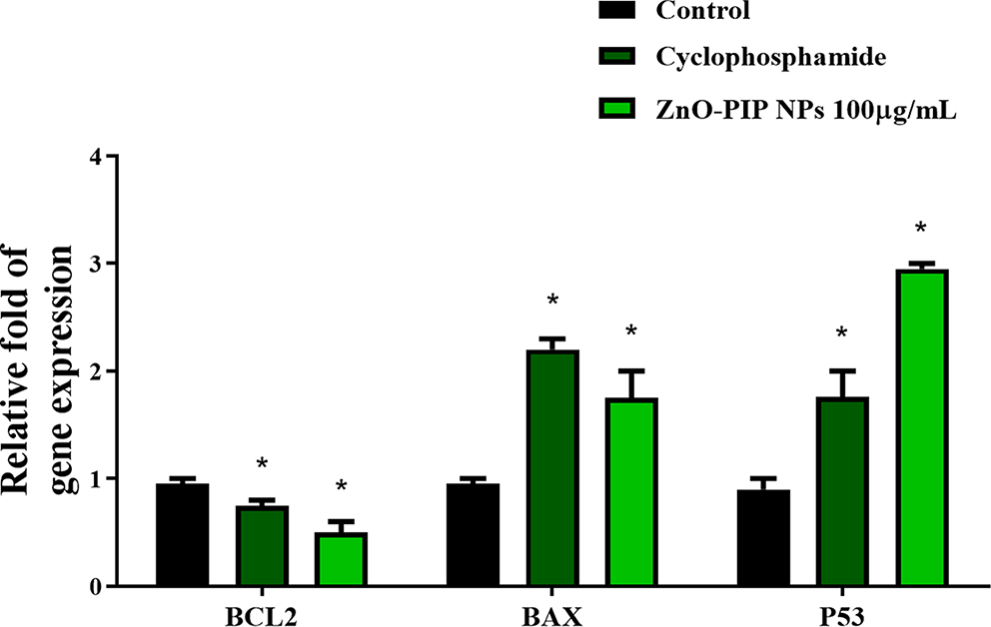
The impact of ZnO-PIP NPs treatment on the mRNA expression levels of BCL2, BAX, and P53 was assessed. Results are presented as the mean ± standard deviation from three separate experiments. Statistical significance (* *p* < 0.05) indicates a comparison with the control group

The examination of ZnO-PIP NPs at a concentration of 100 µg/mL showcased superior antioxidant, antimicrobial, and antiapoptotic properties in comparison to other concentrations. This concentration was deemed optimal and subsequently employed in gene expression studies. The effective anticancer activity involves the downregulation of BCL2 and the upregulation of BAX and P53 gene expression levels. Consistent with these findings, our investigation revealed that KB cells treated with ZnO-PIP NPs at 100 µg/mL displayed a notable decrease (*p* < 0.05) in BCL2 expression (0.4 fold), alongside a simultaneous upregulation of BAX (1.8 fold) and P53 (2.9 fold) levels relative to the control group (Fig. 10).

## Discussion

The combination of ZnO NPs with PIP, a bioactive compound derived from black pepper, presents a promising approach for combating both dental pathogens and oral cancer. ZnO NPs have demonstrated potent antimicrobial properties against a wide range of dental pathogens due to their ability to induce oxidative stress and disrupt microbial cell membranes [21]. Additionally, ZnO NPs have shown potential in inhibiting biofilm formation, a crucial factor in the pathogenesis of dental infections [30]. PIP, on the other hand, possesses antimicrobial properties and has been reported to enhance the bioavailability and efficacy of various drugs [31]. The synergistic action of ZnO NPs and PIP can enhance the antimicrobial activity against dental pathogens by targeting multiple pathways involved in microbial growth and biofilm formation. The combination of these compounds could potentially offer a dual therapeutic approach against oral cancer, inhibiting cancer cell growth while also targeting oral pathogens that may contribute to cancer development or progression.

The oral cavity is subject to oxidative stress due to various factors such as bacterial infections, inflammation, and exposure to reactive oxygen species (ROS) generated during metabolic processes. Antioxidants help neutralize ROS, thus protecting oral tissues from oxidative damage [32]. Antioxidants can be incorporated into dental materials such as composites, cements, and sealants to improve their stability, durability, and biocompatibility. This can extend the lifespan of dental restorations and reduce the risk of secondary complications [33]. Recent research has reported that the antioxidant activity observed in *Piper nigrum* extract primarily originates from its constituent compound, PIP. PIP demonstrates the ability to effectively inhibit or neutralize free radicals and reactive oxygen species. Furthermore, it exerts a positive influence on cellular thiol status, antioxidant molecules, and antioxidant enzymes when studied in vitro [34]. The successful synthesis of ZnO-PIP NPs was confirmed through comprehensive characterization, which provided insights into the morphology and surface features of the NPs, revealing their size distribution and shape. Previous studies have shown that the PIP from *Capsicum chinense* showed the antioxidant acivity [34]. Meanwhile, the ZnO-PIP NPs exhibited superior free radical scavenging activity compared to other tested substances in both the DPPH and ABTS assays. This suggests a potential application of ZnO-PIP NPs in dental materials. Furthermore, the antioxidant activity of ZnO-PIP NPs may also have a beneficial effect on oral health by mitigating oxidative stress in the surrounding oral tissues. This could potentially aid in the prevention or management of dental caries, where oxidative stress plays a significant role in their pathogenesis.

In dental problems, pathogens such as *S. aureus*, *S. mutans*, *E. faecalis*, and *C. albicans* are notorious for their ability to form biofilms, which contribute significantly to oral diseases. Candida species, particularly *C. albicans*, are opportunistic fungi that form biofilms on oral mucosal surfaces, prosthetic devices, and dental implants, leading to oral candidiasis and complications in immunocompromised individuals [35]. *S. mutans*, a primary etiological agent of dental caries, readily forms biofilms on tooth surfaces, producing acid and facilitating the demineralization of enamel. *S. aureus*, a common pathogen associated with oral infections and periodontal diseases, can form biofilms on dental implants and oral tissues, exacerbating inflammatory responses and tissue damage [36]. *E. faecalis*, frequently implicated in persistent endodontic infections and root canal treatment failures, is adept at biofilm formation within the root canal system, rendering it resistant to antimicrobial therapies and promoting treatment resistance. These pathogens’ ability to form robust and resilient biofilms underscores the challenges in managing dental infections and highlights the importance of developing strategies to disrupt biofilm formation for effective treatment and prevention of oral diseases [37]. Recent studies have showed that PIP displays notable antibiofilm efficacy against *S. aureus* through a mechanism involving the accumulation of ROS [38]. Meanwhile, in accordance with the previous result, our study showed the consistent MIC values and substantial zones of inhibition exhibited by ZnO-PIP NPs against dental pathogens were observed, emphasizing their potential as effective antimicrobial agents in dental applications. Dental pathogens utilize receptor proteins as key components in the formation of biofilms, complex microbial communities encased in an extracellular matrix. These receptor proteins play crucial roles in initial attachment, aggregation, and biofilm maturation [39]. *S. aureus* can adhere to tooth surfaces or dental implants, aided by *S. aureus* surface protein G (SasG), which mediates initial attachment to host tissues. Once adhered, *S. aureus* secretes extracellular polymeric substances, forming a biofilm matrix that shields bacteria from host defenses and antimicrobial agents, thus promoting persistent colonization and infection [40]. *S. mutans*, a primary contributor to dental caries, employs its Antigen I/II (AgI/II) protein’s carboxy-terminus to effectively form biofilms and exacerbate dental problems. The carboxy-terminus of AgI/II acts as an adhesin, facilitating the initial attachment of *S. mutans* to tooth surfaces by binding to salivary proteins and the acquired enamel pellicle [41]. Once attached, *S. mutans* secretes glucans using glucosyltransferase enzymes, forming an extracellular matrix that encapsulates bacterial cells, promoting cohesion and biofilm maturation. This dense biofilm structure provides protection against mechanical forces, antimicrobial agents, and host immune responses, enabling *S. mutans* to persistently colonize dental surfaces and metabolize fermentable carbohydrates, leading to acid production and subsequent enamel demineralization, ultimately resulting in dental caries [42]. *E. faecalis*, a common opportunistic pathogen in endodontic infections, utilizes its Enterococcal surface protein (Esp) to facilitate biofilm formation and contribute to dental problems. Esp acts as a surface adhesin, enabling initial attachment to host tissues and dental surfaces, crucial for biofilm initiation. Once adhered, *E. faecalis* secretes extracellular polymeric substances (EPS), forming a protective matrix around bacterial cells within the biofilm. This matrix enhances cohesion and provides resistance to antimicrobial agents and host immune responses, promoting persistent colonization and infection within the root canal system [43]. *C. albicans*, a prevalent fungal species in oral microbiota, employs its Als3 adhesin to form biofilms and exacerbate dental problems. Als3 plays a pivotal role in the initial attachment of *C. albicans* to oral surfaces, including tooth enamel and mucosal tissues, by binding to host cell receptors such as E-cadherin and fibronectin. Once adhered, *C. albicans* secretes extracellular matrix components, including proteins, polysaccharides, and extracellular DNA, forming a robust biofilm structure. This biofilm provides protection against host immune responses and antifungal agents, promoting persistent colonization and contributing to oral diseases such as denture stomatitis and oral candidiasis [44]. The potential interaction between PIP and the receptors involved in biofilm formation among various dental pathogens was evaluated. The results of the study revealed that PIP exhibited a notable affinity towards all the receptors investigated. This finding supports the results obtained from the zone of inhibition assays and biofilm inhibition assays. The outcomes demonstrated that PIP effectively inhibited the growth of dental pathogens and significantly reduced biofilm formation. These results suggest that PIP could be a promising candidate for developing therapeutic agents aimed at disrupting biofilm formation and combating dental infections caused by pathogens such as *S. mutans*, *S. aureus*, *E. faecalis*, and *C. albicans*. Moreover, the ability of PIP to target multiple receptors associated with biofilm formation underscores its potential as a broad-spectrum antimicrobial agent for dental care applications.

The infection by dental pathogens can potentially contribute to the development of oral cancer through various mechanisms. Chronic inflammation triggered by these pathogens’ presence and their byproducts can lead to sustained immune responses, promoting tissue damage and genetic alterations conducive to carcinogenesis [13]. For instance, *S. mutans* produces lactic acid as a byproduct of sugar metabolism, creating an acidic microenvironment that can damage oral epithelial cells and promote their malignant transformation [45]. Additionally, certain pathogens like *C. albicans* can produce carcinogenic compounds or toxins, contributing to oncogenic processes. Moreover, these pathogens are often found within biofilms, which not only shield them from host immune responses but also promote local tissue invasion and metastasis, facilitating the spread of malignant cells. Furthermore, chronic infections can disrupt the oral microbiota balance, leading to dysbiosis, which has been associated with an increased risk of oral cancer. Collectively, the interactions between dental pathogens and host tissues can initiate and perpetuate a pro-inflammatory and pro-carcinogenic milieu within the oral cavity, ultimately predisposing individuals to the development of oral cancer [46]. ZnO-PIP NPs, known for their potent antimicrobial activity against dental pathogens, were further investigated for their potential anticancer effects specifically targeting oral cancer. The results of the study revealed promising anticancer activity, demonstrating a significant reduction in the viability of KB cells, a human oral cancer cell line. This finding suggests that ZnO-PIP NPs possess dual functionality, exhibiting both antimicrobial and anticancer properties. BCL2, BAX, and P53 are pivotal regulators of cellular apoptosis, a process central to cancer development and progression. In oral cancer, dysregulation of apoptosis-related genes such as BCL2, which inhibits apoptosis, and BAX, which promotes apoptosis, can disrupt the delicate balance between cell proliferation and cell death, leading to uncontrolled tumor growth. Additionally, P53, a tumor suppressor gene, plays a crucial role in orchestrating cellular responses to DNA damage and cellular stress, including the induction of apoptosis in damaged or aberrant cells. Given their critical roles in regulating cell fate decisions, alterations in the expression or activity of these genes can significantly influence the development, progression, and response to therapy in oral cancer. Therefore, studying the expression levels and activity of BCL2, BAX, and P53 in response to ZnO-PIP NPs treatment provides valuable insights into the underlying mechanisms of their anticancer effects and elucidates potential therapeutic targets for combating oral cancer. In the study investigating the anticancer activity of ZnO-PIP NPs in KB cells, the results revealed significant alterations in the expression levels of key apoptosis-related genes, including BCL2, BAX, and P53. Treatment with ZnO-PIP NPs resulted in a remarkable downregulation of BCL2, an anti-apoptotic protein known to inhibit cell death, thereby shifting the balance towards apoptosis induction. Concurrently, there was an upregulation of BAX, a pro-apoptotic protein that promotes apoptosis by facilitating mitochondrial outer membrane permeabilization and subsequent cytochrome c release, further enhancing the apoptotic response in KB cells. Moreover, ZnO-PIP NPs treatment led to the upregulation of P53, a tumor suppressor gene crucial for orchestrating cellular responses to DNA damage and stress. The upregulation of P53 likely contributed to the activation of downstream apoptotic pathways, including the transcriptional regulation of pro-apoptotic genes and the induction of cell cycle arrest, ultimately leading to the inhibition of KB cell proliferation and the promotion of cell death. Collectively, these results suggest that the anticancer activity of ZnO-PIP NPs in KB cells is mediated through the modulation of BCL2, BAX, and P53 expression levels, highlighting their potential as promising therapeutic agents for oral cancer treatment by targeting key apoptotic pathways.

Several important considerations warrant discussion regarding the clinical translation of our findings and avenues for future research. Firstly, elucidating the safety profile and biocompatibility of ZnO-PIP NPs is essential for their clinical use. Conducting comprehensive preclinical studies, including in vivo toxicity assessments and biodegradation studies, will be crucial to ensure the safety and tolerability of these nanoparticles in oral applications. Furthermore, exploring alternative delivery methods for ZnO-PIP NPs, such as topical gels, mouthwashes, or dental implants coated with ZnO-PIP NPs, could enhance their accessibility and efficacy in clinical settings. Investigating the stability and release kinetics of ZnO-PIP NPs from these delivery systems will be imperative to optimize their therapeutic outcomes. In addition to their antimicrobial and anticancer properties, the antioxidant activity of ZnO-PIP NPs presents opportunities for mitigating oxidative stress-related oral diseases, including dental caries and periodontal diseases. Future research could focus on evaluating the potential of ZnO-PIP NPs in promoting oral tissue regeneration and wound healing, particularly in the context of periodontal therapy and oral surgery.

## Conclusion

In conclusion, this research demonstrates the multifunctional capabilities of ZnO-PIP NPs in addressing oral health challenges, including microbial infections and oral cancer. Through their potent antimicrobial and anticancer activities, ZnO-PIP NPs exhibit promising potential as integrated therapeutic agents for oral healthcare interventions. The robust findings from this study provide a solid foundation for the continued exploration and development of ZnO-PIP NPs, offering new avenues for improved management and treatment of oral diseases. These NPs hold promise for advancing oral healthcare practices, ultimately benefiting patients by enhancing treatment outcomes and quality of life.

### Electronic supplementary material

Below is the link to the electronic supplementary material.

Supplementary Material 1

## Data Availability

The data are available from the corresponding author upon reasonable request.
