# Supplementary material for: Piperine-coated zinc oxide nanoparticles target biofilms and induce oral cancer apoptosis via BCl-2/BAX/P53 pathway
Source: BMC Oral Health. 2024 Jun 21;24:715. doi: 10.1186/s12903-024-04399-z (PMC11191213; doi:10.1186/s12903-024-04399-z)
Supplement: Supplementary file 1 — Supplementary Material 1 [file 12903_2024_4399_MOESM1_ESM.docx]

E-Supplementary Table 1: List of abbreviations

| Piperine | PIP |
| --- | --- |
| Zinc oxide | ZnO |
| Nanoparticles | NPs |
| Scanning Electron Microscope | SEM |
| Reactive Oxygen Species | ROS |
| Computed Tomography | CT |
| Magnetic Resonance Imaging | MRI |
| X-ray Diffraction analysis | XRD |
| Fourier-transform infrared spectroscopy | FTIR |
| Energy dispersive X-ray analysis | EDAX |
| 2,2-diphenyl-1-picrylhydrazyl | DPPH |
| 2,2′-azino-bis-(3-ethylbenzothiazoline-6-sulfonic) | ABTS |
| Minimal Inhibitory Concentration | MIC |
| Human epithelial carcinoma cells | KB cells |
| 3-(4,5-dimethylthiazol-2-yl)-2,5-diphenyltetrazolium bromide | MTT |
| Phosphate-buffered saline | PBS |
| Lysine | LYS |
| Glycine | GLY |
| Alanine | ALA |
| Arginine | ARG |
| Valine | VAL |
| Isoleucine | ILE |
| Serine | SER |
